# Biological Maturity Status Strongly Intensifies the Relative Age Effect in Alpine Ski Racing

**DOI:** 10.1371/journal.pone.0160969

**Published:** 2016-08-09

**Authors:** Lisa Müller, Erich Müller, Carolin Hildebrandt, Christian Raschner

**Affiliations:** 1 Department of Sport Science, University of Innsbruck, Innsbruck, Tyrol, Austria; 2 Department of Sport Science and Kinesiology, University of Salzburg, Salzburg, Salzburg, Austria; Norwegian University of Science and Technology, NORWAY

## Abstract

The relative age effect (RAE) is a well-documented phenomenon in youth sports. This effect exists when the relative age quarter distribution of selected athletes shows a biased distribution with an over-representation of relatively older athletes. In alpine ski racing, it exists in all age categories (national youth levels up to World Cup). Studies so far could demonstrate that selected ski racers are relatively older, taller and heavier. It could be hypothesized that relatively younger athletes nearly only have a chance for selection if they are early maturing. However, surprisingly this influence of the biological maturity status on the RAE could not be proven, yet. Therefore, the aim of the present study was to investigate the influence of the biological maturity status on the RAE in dependence of the level of competition. The study investigated 372 elite youth ski racers: 234 provincial ski racers (P-SR; high level of competition) and 137 national ski racers (N-SR; very high level of competition). Anthropometric characteristics were measured to calculate the age at peak height velocity (APHV) as an indicator of the biological maturity status. A significant RAE was present among both P-SR and N-SR, with a larger effect size among the latter group. The N-SR significantly differed in APHV from the P-SR. The distribution of normal, early and late maturing athletes significantly differed from the expected normal distribution among the N-SR, not among the P-SR. Hardly any late maturing N-SR were present; 41.7% of the male and 34% of the female N-SR of the last relative age quarter were early maturing. These findings clearly demonstrate the significant influence of the biological maturity status on the selection process of youth alpine ski racing in dependence of the level of competition. Relatively younger athletes seem to have a chance of selection only if they are early maturing.

## Introduction

To guarantee fair competition and reflect age-related development, the youth athletes in many sports are separated into competition categories by chronological age [1–4]. Often, January 1^st^ is used as the cut-off date for each selection year [1; 3; 5]. Athletes born in the same year compete against each other in the same competition category. Although this strategy is well intentioned, it is responsible for creating chronological age advantages; age differences of almost 12 months are possible between individuals [3; 6]. In youth sports, these differences have lead to the already well-documented *relative age effect* (RAE) phenomenon. A RAE exists when the relative age quarter distribution (relative age quarter corresponds to the birth quarter) of selected athletes is not equally distributed among the four quarters, as the distribution of the general population is [7–8], but it shows a skewed distribution with an over-representation of relatively older athletes, whose birth months are close to the cut-off date for the competition categories [3; 7].

The RAE is a problem in various types of sport; its presence was first documented in Canadian ice hockey [6], and since then, it has been investigated in many other sports, like soccer, basketball, tennis a.o., as well [1; 7]. From an ethical point of view, the existence of a RAE indicates that the talent development systems in the affected types of sport seem to be biased against relatively younger athletes. This leads to the fact that relatively younger athletes have fewer opportunities of reaching the elite level despite their talents and efforts and that many talented, relatively younger athletes often drop out of a sport early and go unnoticed [9–10]. Because talent in a sport does not depend on the birth month [5], it can be assumed that there is a severe loss of talent due to the existence of a RAE in these sports.

The RAE is also a problem in alpine ski racing in all age categories and at both national and international levels [9–11]. It starts to exist already at the youngest levels of national youth ski in Austria [10; 12] and Switzerland [13]. The effect continues to be present in alpine ski racing at international youth levels; it was evident at the 1^st^ winter Youth Olympic Games (YOG) in 2012 [8], the 12^th^ winter European Youth Olympic Festival in 2015 in Austria and Liechtenstein [14] (14 to 15 years), and the 2009 to 2011 FIS Junior World Ski Championships (16 to 20 years) [15]. The RAE in alpine ski racing is not only present at national and international youth levels but also at the elite level as it was observed at the FIS World Cup [15–16]. The existence of the RAE in all age categories of alpine ski racing leads to the assumption that there is a severe loss of talent [10] because talent in a sport does not depend on the birth month [5] and therefore, the skewed relative age quarter distributions indicate that many talented relatively younger ski racers did not get the chance to reach the elite level [10]. Often talent identification systems promote selection biases that confuse maturation for talent. This maturation hypothesis proposed by Baker et al. [16] to explain RAE in sports, is based on the assumption that the relative age of an athletes is related to his/her cognitive and physical maturation. Hence, maturational differences between relatively older athletes (born early in selection year) and relatively younger athletes (born late in the selection year) influence the selection of youth athletes. [16–17] As a consequence, in the short-term, relatively older and earlier maturing athletes are favourably selected as potentially “more talented” and relatively younger and less mature athletes are often overlooked and excluded [18–19]. Relatively older athletes have an increased likelihood of advanced physical characteristics and of entering puberty earlier compared to relatively younger athletes of the same competition category. Additionally, in sports with high demands on strength and power, youth athletes have been identified above average for height and weight compared to non-athletes of the same age. [1] Hence, the combination of a relatively older age and an advanced physical maturation leads to a selection advantage and as a consequence, to the RAE. [1; 16–18]

In many different sports, e.g., handball and basketball [20], RAEs often do not occur among female athletes. In other sports, such as in soccer [21], RAEs are indeed also present among female athletes. However, the general trend of different sports shows a stronger RAE among male athletes. This is in line with studies of alpine ski racing, in which a stronger RAE was found among male athletes at youth levels [10] and international youth and elite levels [15–16].

A RAE can be better explained using a domain-specific model, which means that the diverse interacting factors that influence the participation rate should be investigated separately for each specific sport [22]. Based on this suggestion, and the fact that alpine ski racing is a sport that requires a high level of physical fitness [23–25] and a sport in which athletes with greater body mass and height have advantages [26], Müller et al. [9] investigated the influential mechanisms of the RAE in alpine ski racing. In this study, the authors additionally included a sample of non-athletes of the same age (10–13 years) and region in order to be able to assess whether possible influential mechanisms are generally valid for this age group of youths or whether they are indeed only valid for youth ski racers. They found out that the talent development system in this sport seems to be influenced by anthropometric characteristics; relatively older ski racers were taller and heavier than relatively younger ski racers and compared with the group of non-athletes. There were no differences in biological maturity status and level of physical performance among the youth ski racers of the four relative age quarters. The authors concluded that the small sample of relatively younger youth ski racers, who were selected despite their relatively younger age, could counteract the relative age disadvantage if they showed the same level of physical fitness and maturity as their relatively older counterparts. Because it has already been shown that athletes with larger anthropometric characteristics have advantages in alpine ski racing [26], it was hypothesized that the ski racers would be more mature than the group of non-athletes of the same age and that the ski racers of the four quarters would differ in their biological maturity status from each other. However, these differences could not be shown, which was surprising [9]. Based on this surprising finding, the authors emphasized the necessity for further research regarding the influence of the biological maturity status on the selection process and, thus, on the RAE in alpine ski racing in dependence of the competition level.

Also in other sports, like in soccer [27] and ice hockey [28], it could be demonstrated that more mature athletes were favourably selected for elite squads on high levels of competition. Based on this, Müller et al. [9] still speculated that the biological maturity status indeed plays a role in the talent selection and development process in alpine ski racing. Therefore, they demanded to assess the influence of the biological maturity status on the RAE in dependence of the level of competition. The authors emphasized the necessity for further research to accurately investigate the role of the biological maturity status in the talent selection process of national youth ski racing. In this context, they demanded to examine a selected sample of the most talented youth ski racers on a very high competition level. [9]

Therefore, the aim of the present study was to investigate the influence of the biological maturity status on the RAE in dependence of the level of competition by comparing a sample of provincial youth ski racers (high competition level) with a sample of youth athletes selected for the national final races (very high competition level) who represent the most talented Austrian ski racers of their given age.

## Materials and Methods

### Participants

Elite youth ski racers from Austrian ski boarding schools (= group of provincial ski racers; high level of competition) and elite youth ski racers who were selected for the national final races (= group of national ski racers; very high level of competition) participated in this study. The mean age of the subjects was 12.2 years (SD = 1.16; range = 9.8–15.4). In total, 372 youth ski racers (201 males, 171 females) were examined: 234 provincial ski racers (131 males, 103 females; mean age: 12.5 ± 1.3; range: 9.8–15.4) and 137 national ski racers (69 males, 68 females; mean age: 11.5 ± 0.6; range: 10.3–12.3). Table 1 presents the anthropometric data (means and standard deviations) for male and female athletes, separated by the two groups of provincial and national ski racers. Additionally, the birth dates of 413 pupils (comparison group) from secondary modern schools or grammar schools in the same regions and who were the same age as the participants were collected.

**Table 1:** Anthropometric characteristics separated by gender and group of ski racers.

	provincial ski racers		national ski racers	
Anthropometric characteristic	male	female	male	female
	*M* (±*SD*)	*M* (±*SD*)	*M* (±*SD*)	*M* (±*SD*)
Age [yrs]	12.3 (±1.2)	12.4 (±1.3)	11.6 (±0.5)	11.5 (±0.6)
Body weight [kg]	44.1 (±9.2)	46.8 (±10.8)	40.5 (±5.1)	40.6 (±6.1)
Body height [cm]	153.6 (±9.4)	155.4 (±9.6)	149.5 (±6.3)	149.8 (±7.3)
Body mass index [kg/m²]	18.5 (±2.2)	19.1 (±2.6)	18.2 (±1.6)	18.0 (±1.5)
Sitting height [cm]	79.0 (±4.3)	80.6 (±4.9)	77.2 (±3.5)	77.6 (±3.4)
Age at peak height velocity [yrs]	13.8 (±0.5)	12.2 (±0.6)	13.6 (±0.4)	11.9 (±0.4)

M = mean; SD = standard deviation.

### Ethics Statement

Parents and participants were informed of the study aims, requirements and risks before they provided written informed consent. The study was performed according to the Declaration of Helsinki. The study was approved by the Institutional Review Board of the Department of Sport Science of the University of Innsbruck and the Board for Ethical Questions in Science of the University of Innsbruck (Nr.: 2/2014).

### Measurements and Procedures

The birth dates of the participants were collected and the athletes were categorized into four relative age quarters according to their birth months. The cut-off date for the competition categories in alpine ski racing is January 1^st^; therefore the months were split into quarters to calculate the relative age quarters as follows: relative age quarter one (Q1) included the months January to March; quarter 2 (Q2) the months April to June; quarter 3 (Q3) the months July to September; and quarter 4 (Q4) the months October to December. The relative age quarter distribution of the comparison group of non-athletes, which was a nearly even distribution among the four quarters (i.e., nearly 25% in each quarter), was used as the expected distribution for the analyses concerning the existence of a relative age effect within the youth ski racers (separated by gender).

The biological maturity status was calculated using the non-invasive method proposed by Mirwald et al. [29]. These prediction equations include the following anthropometric parameters, which were assessed according to previously described procedures [30]: body mass (0.1 kg, Seca, Hamburg, Germany), body height (0.1 cm, Seca Portable Stadiometer, Hamburg, Germany) and sitting height (0.1 cm, Seca Portable Stadiometer, Hamburg, Germany; sitting height table). Additionally, the calculations of leg length as the difference between body height and sitting height, and actual chronological age at the time of measurement are included in the equations. These parameters were used to predict maturity offset, the time before or after peak height velocity (PHV), using the prediction equations from Mirwald et al. [29]. The predicted age at peak height velocity (APHV) was then calculated as the difference between chronological age and maturity offset. The validity of this method was previously proven for this type of participant, i.e., elite youth ski racers and non-athletes of the same age as the participants in the present study. In the validity-study the APHV-method was compared with the x-rays of the left wrist and a good validity could be assessed. [31] The APHV-method was recently used in several studies including youth athletes [32–33]. Following the approach by Deprez et al. [33], the participants were then divided into three maturity groups (late, normal and early maturing) based on the mean (M) ± standard deviation (SD) of the APHV of the provincial ski racers (separated by gender) because the provincial ski racers represented the sample from which the national ski racers were selected. An athlete was classified as normal maturing if his or her APHV was within M ± SD, early maturing if his or her APHV was less than M—SD, and late maturing if his or her APHV was higher than M + SD. In the validity-study it could be shown that the categorization of normal, early and late maturing athletes based on the APHV-method did not significantly differ from the categorization based on the skeletal age [31].

### Statistical Analysis

To assess the differences between the observed and the expected relative age quarter distributions, chi²- tests (χ²) were used for the total sample and for samples separated by gender and by group of ski racers (national vs. provincial). The expected distribution was the distribution of the comparison group of non-athletes. The effect size ω was calculated for the χ²- tests, as proposed by Wattie et al. [22]. Odds ratios (*OR*) and 95% confidence intervals (*95% CI*) were calculated for the relative age quarter distribution of the total sample and according to gender and the group of ski racers, as proposed by Cobley et al. [1].

The normal distribution of the APHV was tested using the Kolmogorov-Smirnov test (separated by gender, group of ski racers and single relative age quarters). To evaluate differences in the APHV between the provincial and national ski racers (separated by gender and relative age quarter), Student’s t-tests were used. To assess the influence of the biological maturity status on the RAE, univariate analyses of variance were used (dependent variable: biological maturity status (APHV); independent variable: relative age quarter). The variance homogeneity was assessed with Levene-Test and for post-hoc-tests, those of Scheffé were used. To evaluate the difference between the expected (normal) distribution of early, normal and late maturing athletes among each relative age quarter and the observed distribution (separated by gender and group of ski racers), χ²- tests were used.

The level of significance was set at *p*<0.05, and for highly significant at *p*<0.01. All of the calculations were performed using PASW Statistics 21.0; the effect size was assessed using G*Power 3.1.9.2.

## Results

The χ²- statistics showed a significant difference between the relative age quarter distribution of the ski racers (provincial ski racers: χ²(3, N = 234) = 8.02; p = 0.046; ω = 0.19; national ski racers: χ²(3, N = 137) = 16.49; p = 0.001; ω = 0.35) compared with that of the comparison group, which showed a nearly equal distribution. A significant difference was also shown between the relative age quarter distribution of the male ski racers (provincial: χ²(3, N = 131) = 9.0; p = 0.029; ω = 0.26; national: χ²(3, N = 69) = 11.29; p = 0.01; ω = 0.41) and that of the male pupils in the comparison group. A significant difference was also shown between the relative age quarter distribution of the female national ski racers (χ²(3, N = 68) = 8.24; p = 0.041; ω = 0.35) and that of the female pupils in the comparison group. No significant difference was present between the relative age quarter distribution of the female provincial ski racers and that of the female pupils (p = 0.65). The relative age quarter distributions of the male and female provincial and national ski racers are presented in Table 2. The descriptive OR and the corresponding χ² for each quarter of the provincial and national ski racers are presented in Table 3. The OR calculations revealed significant differences in the total sample of provincial ski racers and among the male provincial ski racers between relative age quarter 1 and 3 and between the first and the last relative age quarter. The likelihood of participation in provincial races of a male athlete of the first relative age quarter was 2.5 times higher than for one of the last quarter. Among the female provincial ski racers no significant differences were found. Among the national ski racers significant differences were found already between athletes of the first and the second relative age quarter for the total sample and the male athletes. Among the female national ski racers, a significant difference was found between athletes of the first and the last relative age quarter with a 4.1 times higher likelihood of selection for female athletes of the first quarter. The likelihood of selection of a male athlete of the first quarter was 3.4 times higher than for one of the second quarter.

**Table 2:** Relative age quarter distributions of provincial and national ski racers according to gender.

		Relative age quarter						
		Q1	Q2	Q3	Q4	*p*	chi²	*ω*
Category		[%]	[%]	[%]	[%]			
Provincial ski racers	Total (n = 234)	32.5	25.2	20.9	21.4	0.046	8.02	0.19
	Male (n = 131)	35.1	26.0	21.4	17.6	0.029	9.00	0.26
	Female (n = 103)	29.1	24.3	20.4	26.2	0.646	1.66	0.17
National ski racers	Total (n = 137)	38.7	23.4	23.4	14.6	0.001	16.49	0.35
	Male (n = 69)	42.0	17.4	23.2	17.4	0.010	11.29	0.41
	Female (n = 68)	35.3	29.4	23.5	11.8	0.041	8.24	0.35

Q1-4 = relative age quarter 1–4; ω = effect size.

**Table 3:** Descriptive OR across all relative age quarters according to group of ski racers and gender.

sample			Q1:Q2	Q1:Q3	Q1:Q4
Provincial ski racers	Total (n = 234)	chi²	2.14	5.83	5.37
		*p* value	0.143	0.016	0.021
		*OR* [95% CI]	1.47 (0.95–2.13)	**1.82 (1.20–2.76)**	**1.77 (1.17–2.68)**
	Male (n = 131)	chi²	1.8	4.38	7.67
		*p* value	0.18	0.036	0.006
		*OR* [95% CI]	1.54 (0.91–2.62)	**1.99 (1.15–3.45)**	**2.54 (1.43–4.52)**
	Female (n = 103)	chi²	0.46	1.59	0.16
		*p* value	0.50	0.208	0.691
		*OR* [95% CI]	1.28 (0.69–2.38)	1.61 (0.85–3.05)	1.16 (0.63–2.13)
National ski racers	Total (n = 137)	chi²	5.19	5.189	14.92
		*p* value	0.023	0.023	<0.001
		*OR* [95% CI]	**2.07 (1.23–3.50)**	**2.07 (1.23–3.50)**	**3.69 (2.05–6.63)**
	Male (n = 69)	chi²	7.05	3.76	7.05
		*p* value	0.008	0.053	0.008
		*OR* [95% CI]	**3.44 (1.57–7.55)**	**2.40 (1.15–5.01)**	**3.44 (1.57–7.55)**
	Female (n = 68)	chi²	0.36	1.6	8.0
		*p* value	0.546	0.206	0.005
		*OR* [95% CI]	1.31 (0.64–2.69)	1.77 (0.84–3.75)	**4.09 (1.68–9.96)**

*OR* = odds ratio; *CI* = confidence interval; Q1-4 = relative age quarter 1–4

bolded values indicate significance of odds ratio (i.e., 95% CI does not include 1).

The male and female national ski racers significantly differed in APHV from the male (*t* = 2.53; p = 0.012) and female provincial ski racers (*t* = 3.97; p<0.001), respectively (Table 1). The national ski racers showed lower values in APHV compared with the provincial ski racers. The male national ski racers showed a mean APHV of 13.62 (±0.38) years; the provincial ski racers, however, will reach their PHV at a mean age of 13.79 (±0.55) years. The female national ski racers will reach their PHV also at a younger mean age of 11.92 (±0.37) years compared with the female provincial ski racers (12.21 ± 0.58 years). The mean values of APHV and the standard deviations separated by gender and group of ski racers are presented separately for the four relative age quarters in Table 4. Differences in the biological maturity status between national and provincial ski racers were shown in both genders but only among athletes of the fourth relative age quarter (male: *t* = 2.41, *p* = 0.022; female: *t* = 2.52, *p* = 0.017). The analyses of variance showed that there was no significant difference in biological maturity status among the male and female ski racers of the four relative age quarters, neither within the group of national ski racers nor within the group of provincial ski racers.

**Table 4:** Biological maturity status of provincial and national ski racers.

Gender		Age at peak height velocity [yrs]		difference provincial vs. national ski racers
		provincial ski racers	national ski racers	
		*M* (±*SD*)	*M* (±*SD*)	
Male athletes	Q1	13.77 (±0.49)	13.68 (±0.43)	n.s.
	Q2	13.74 (±0.64)	13.65 (±0.37)	n.s.
	Q3	13.80 (±0.59)	13.57 (±0.31)	n.s.
	Q4	13.88 (±0.47)	13.51 (±0.35)	*t* = 2.41; *p* = 0.022
Female athletes	Q1	12.22 (±0.62)	11.93 (±0.44)	n.s.
	Q2	12.17 (±0.58)	11.91 (±0.39)	n.s.
	Q3	12.21 (±0.58)	11.94 (±0.37)	n.s.
	Q4	12.24 (±0.56)	11.91 (±0.24)	*t* = 2.52; *p* = 0.017

M = mean; SD = standard deviation; Q1-4 = relative age quarter 1–4; n.s. = not significant.

The provincial and national ski racers were categorized as normal, early or late maturing based on the mean and standard deviation of the APHV of the provincial ski racers. Among the male athletes, an athlete was normal maturing if he had an APHV between 13.34 and 14.33 years, early maturing if he had an APHV younger than 13.34 years, and late maturing if he had an APHV higher than 14.33 years. A female athlete was categorized as normal maturing if she had an APHV between 11.63 and 12.79 years, early maturing if she had an APHV lower than 11.63 years, and late maturing if she had an APHV greater than 12.79 years.

The distribution of normal, late and early maturing athletes nearly corresponded to the expected normal distribution among the provincial ski racers (normal: 72.2%; early: 12.8%; late: 15.0%); there was no significant difference between the expected normal distribution and the distribution of normal, early and late maturing provincial ski racers (p = 0.368). The distribution of both male (normal: 71.8%; early: 13.7%; late: 14.5%) and female (normal: 72.8%; early: 11.7%; late: 15.5%) provincial ski racers also did not significantly differ from the expected normal distribution (male: p = 0.69; female: p = 0.48). The distribution of normal, early and late maturing national ski racers significantly differed from the expected normal distribution (χ²(3, N = 137) = 21.29; p<0.001; ω = 0.40). Most of the athletes (79.6%) were normal maturing, 19% were early maturing and 1.4% were late maturing. The distribution of normal, early and late maturing athletes also significantly differed from the expected normal distribution for both male (normal: 81.2%, early: 17.4%, late: 1.4%; χ²(3, N = 69) = 10.80; p = 0.005; ω = 0.40) and female national ski racers (normal: 77.9%, early: 20.6%, late: 1.5%; χ²(3, N = 68) = 10.76; p = 0.005; ω = 0.39). The percentages of normal, early and late maturing athletes separated by group of ski racers, gender, and relative age quarter are presented in Table 5.

**Table 5:** Percentages of normal, early and late maturing athletes separated by relative age quarter.

		Relative age quarter							
Gender		Q1		Q2		Q3		Q4	
		P-SR	N-SR	P-SR	N-SR	P-SR	N-SR	P-SR	N-SR
		[%]	[%]	[%]	[%]	[%]	[%]	[%]	[%]
Male athletes	normal	73.9	93.1	64.7	75.0	71.4	81.3	78.3	58.3
	early	10.9	6.9	17.6	16.7	21.4	18.8	4.3	41.7
	late	15.2	0	17.6	8.3	7.1	0	17.4	0
Female athletes	normal	76.7	70.8	68.0	85.0	76.2	75.0	70.4	66.0
	early	10.0	25.0	20.0	15.0	9.5	25.0	7.4	34.0
	late	13.3	4.2	12.0	0	14.3	0	22.2	0

Q1-4 = relative age quarter 1–4; P-SR = provincial ski racers; N-SR = national ski racers.

## Discussion

The RAE is a problem in all age categories of alpine ski racing [11]. Many young, talented athletes go unnoticed and often drop out of the sport early because they had a late birth month and their maturation maybe was delayed. As a consequence, there is a severe loss of talented youth ski racers because talent in a sport does not depend on the birth month [5] and therefore, the skewed relative age quarter distributions indicate that many talented relatively younger ski racers did not get the chance to reach the elite level [10–11]. The importance of changing strategies in the talent development system in alpine ski racing to minimize the RAE in the future has already been emphasized [9]. However, the underlying factors influencing the RAE in alpine ski racing are not absolutely clear.

Müller et al. [9] showed that relatively older athletes have an additionally increased likelihood of selection if they are taller and heavier. Moreover, it seems that relatively younger athletes nearly only have a chance of selection for elite squads, despite their relative age disadvantage, if they are at the same level of physical performance as the relatively older athletes and if they are early maturing. Relatively older athletes, however, have an increased likelihood of selection independent of their maturity status because they are selected although they might be normal or late maturing. Müller et al. [9] emphasized the necessity to further research the influence of the biological maturity status on the selection, and thus, on the RAE among a selected sample of the most talented youth ski racers. Therefore, the aim of the present study was to investigate the influence of the biological maturity status on the RAE in dependence of the competition level by comparing a sample of provincial youth ski racers with a sample of youth athletes selected for the national final races.

As observed in previous literature [9–16], it was not surprising that the present study reported a significant RAE with an over-representation of relatively older athletes in the total samples of provincial and national youth ski racers. A higher competition level (national versus provincial races) was associated with a stronger RAE because the RAE was even more pronounced among the athletes selected for the national final races; the national ski racers had a larger effect size (ω = 0.35) compared with the provincial ski racers (ω = 0.19). Separated by gender, a similar trend was present. This is in line with the results from Müller et al. [10], in which a stronger RAE was present among athletes selected for national races compared athletes at provincial levels. This trend seems to be especially pronounced in alpine ski racing because studies of other sports, such as handball [34], showed that the strength of the RAE decreased as the competition level increased; this indicates that early development processes are more germane for stronger RAEs. However, it must be considered that in handball, a higher competition level is associated with older players and not with an additional selection for a higher competition level, such as in alpine ski racing. This means that the selection of 10- to 12-year-old ski racers for national final races additionally increases the RAE. The ORs calculations clearly demonstrate the bias of the selection process to identify the most talented youths. The likelihood of selection for a youth ski racer of the first relative age quarter was 3.7 times higher than that for a comparable athlete of the last relative age quarter.

The results of the present study regarding gender specific differences in the occurrence and strength of the RAE are in line with the findings of other studies in alpine ski racing in which a stronger RAE was found among male athletes at youth levels [10] and international youth and elite levels [15–16]. The results of the present study showed that among youth ski racers of provincial races, no significant RAE was present among female athletes; however, a significant RAE was found among male athletes. At the national level, the RAE was present for both male and female athletes with the same large effect size (ω = 0.41). However, the OR calculations revealed that male national ski racers of the first relative age quarter had a 3.4 times greater likelihood of selection already compared with those of the second relative age quarter. Whereas, at the provincial level, significant differences were present only between male athletes of the first and both those of the third and fourth relative age quarter; however, the differences were not that large, ranging from 2 to 2.5. Among female athletes, a significant difference was present only at the national level; those of the first relative age quarter were 4.1 times more likely to be selected compared with those of the last relative age quarter. These values are comparable to those from Müller et al. [10], in which athletes of the first relative age quarter had a 4.7 times higher likelihood of selection for the national final races compared with those of the last quarter.

In accordance with previous studies in alpine ski racing [9] and soccer [32–33], the results of the present study revealed that there were no significant differences in APHV among the youth ski racers of the four relative age quarters. This means that the youth ski racers will reach their individual peak growth spurt at nearly the same age, independent of their relative age quarter. Additionally, it seems that relatively younger athletes can counteract their relative age disadvantages if they are at the same biological maturity status as the relatively older athletes [9]. However, the findings of the present study revealed that both the male and female national ski racers significantly differed in APHV from the male and female provincial ski racers, respectively. The national ski racers will reach their individual peak growth spurt at a significantly younger age than the provincial athletes. This means that more mature athletes were favourably selected for the national final races. This is in line with previous studies of other sports, such as soccer [28], ice hockey [35] and basketball [36], in which selected athletes for elite squads were taller, heavier and more mature than non-selected athletes.

Sherar and colleagues [35] additionally assessed that with every 1-month increase in APHV, adolescent male ice hockey players became 17% less likely to be selected for competitive squads. On average, the male provincial ski racers will reach their individual peak growth spurt nearly 2 months later than the male national ski racers. Among the female athletes, the national ski racers will reach their PHV on average 3.5 months earlier than the provincial athletes. This indicates that more mature athletes indeed have advantages in the selection for national races in youth alpine ski racing.

However, a more detailed analysis showed that the difference in APHV between provincial and national ski racers was significant only among athletes of the fourth relative age quarter for both male and female ski racers. The male national ski racers of Q4 will reach their PHV on average 4.4 months earlier than the provincial ski racers of Q4. Similarly, the female national ski racers of Q4 will reach their PHV on average 4.0 months earlier than the provincial athletes. These results indicate that athletes of the last relative age quarter who were selected for the national final races differed in their biological maturity status from those who were not selected. As a consequence, the biological maturity status significantly influenced the selection process of youth alpine ski racing.

As performed in the study by Deprez et al. [33], the athletes in the present study were divided into three groups of maturity status: normal, early and late maturing athletes. The distribution of normal, early and late maturing provincial ski racers did not significantly differ from the expected normal distribution, neither among the male nor among the female athletes. In contrast, the distribution of the national ski racers significantly differed from the expected normal distribution for the total sample (ω = 0.40) and for both male (ω = 0.40) and female athletes (ω = 0.39). The analyses revealed that among the national ski racers, hardly any late maturing athletes were present (total sample: 1.4%; male: 1.4%; female: 1.5%). The majority of the athletes were normal maturing (total: 79.6%) and 19% were early maturing, which is a higher percentage than expected. A similar distribution was present among male and female athletes; 17.4% and 20.6% were early maturing, respectively. These results emphasize the previously mentioned findings that the biological maturity status plays an important role in the talent selection process of alpine ski racing because late maturing athletes seem to be rarely selected for national final races.

Additionally, the more detailed analyses concerning the distribution of normal, early and late maturing athletes (separated by relative age quarter) revealed that the percentage of early maturing male national ski racers increased from relative age quarter 1 to 4. Late maturing athletes were present only in the second relative age quarter. Almost half (41.7%) of the male national ski racers of the last relative age quarter were early maturing. These values clearly demonstrate that a relatively younger athlete will only be selected for national final races if he is early maturing, whereas athletes of the first and second relative age quarter seem to have a better chance of selection independent of their maturity status. A similar result was also found among the female athletes. Late maturing female national ski racers were only found in the group of relatively older athletes, i.e., those of Q1. The percentage of early maturing athletes ranged between 15% and 34% and that of the last relative age quarter (34%) was only slightly smaller than the percentage of early maturing male athletes (41%). However, as early maturing athletes constituted more than a third of the athletes, the percentage was still high. Thus, female athletes of the last relative age quarter also seem to counteract the relative age quarter disadvantage if they are early maturing. These results are, for the most part, in line with the soccer study by Deprez et al. [33]. They demonstrated that early maturing athletes were overrepresented in the last relative age quarter, whereas late maturing athletes were overrepresented in the first relative age quarter. They concluded that athletes of the last relative age quarter may have an increased chance of selection if they reach their PHV at an earlier age, whereas athletes of the first relative age quarter have an increased chance of selection independent of their maturity status. A similar trend was also found in the present study, even though the maturity status seemed to be a stronger influential factor for selection in alpine ski racing than in soccer because among the athletes selected for the national races, hardly any late maturing athletes were present in all relative age quarters. However, the high percentage of early maturing youth ski racers of the last relative age quarter clearly demonstrates the selection bias in dependence of the level of competition. Relatively older athletes clearly have an advantage in alpine ski racing nearly independent of their maturity status, whereas relatively younger athletes have to be more mature and early maturing to be selected.

## Limitations

The method of calculating the age at peak height velocity as an indicator of the biological maturity status may not be the most accurate one and the authors of the study recognize this as a limitation of the study. Malina et al. [37] showed in a longitudinal study that the predicted APHV varied with chronological age at prediction especially among early and late maturing athletes. However, they did not compare the APHV-method with the estimation of skeletal age. Therefore, this comparison was conducted in a sample of youth ski racers and non-athletes of the same age as the participants of the present study. The validity of the APHV-method could be proven by comparing the x-ray of the left wrist with the APHV-method [31]. In this study, the concordance of the skeletal age and the APHV could be shown also for early and late maturing athletes. Therefore, the APHV-method was used in the present study.

Additionally, the categorization in normal, early and late maturing athletes can be recognized as a limitation of the study. This new approach may differ from the categorization of normal, early and late maturity based on more precise methods like the skeletal age. However, this categorization was done based on previous studies by Deprez et al. [33]. Additionally, in the validity-study, in which the APHV-method and the x-ray of the left wrist were compared, no significant differences could be shown in the categorization into normal, early and late maturing athletes based on the two methods [31].

Another limitation of the study was the broad age range of the participants, especially considering the APHV calculations, because Malina et al. [37] revealed that the dependence of the APHV upon chronological age has implications for studies combining athletes of broad age ranges. However, the age range was that broad only among the provincial ski racers, not among the national ski racers.

## Conclusion

The results of the present study indicate that the RAE is a severe problem in national youth ski racing. The higher the level of youth ski racing (national vs. provincial races), the stronger the RAE is. The youth ski racers selected for national final races significantly differed in their biological maturity status from the provincial ski racers; the latter group will reach their individual peak growth spurt at a significantly older age. The ski racers of the four relative age quarters did not significantly differ in the biological maturity status from each other. In addition, the distribution of normal, early and late maturing athletes clearly at the provincial level was not significantly different from the expected normal distribution; however, at the national level, the distribution significantly differed. Among the athletes selected for national races, hardly any late maturing athletes were present, and among the athletes of the last relative age quarter, there was a high percentage of early maturing athletes. These findings clearly demonstrate the significant influence of the biological maturity status on the selection process of youth alpine ski racing in dependence of the level of competition: the higher the level of competition, the higher the influence of the biological maturity status. Relatively younger athletes seem to have a chance of selection only if they are early maturing. Because the selection for national races is a very important step in the talent development system of alpine ski racing in Austria, strategies in this process should be changed to guarantee greater fairness, to prevent selection errors that disadvantage relatively younger and late maturing athletes and to prevent high drop-out rates of highly talented youth athletes. It can be assumed that the higher the level of youth ski racing, the higher the influence of biological maturation on the selection process, and hence, on the RAE is present. Based on the findings of the present study, the relative age quarter and the biological maturity status should be considered in the talent selection process of alpine ski racing. Till et al. [38] showed that the process of favourably selecting relatively older and early- maturing athletes within competitive youth sports may be counterproductive in the long term because it may exclude equally skilled individuals from opportunities to reach the elite level due to their delayed physical characteristics compared with their relatively older counterparts.
